# Machine learning assisted classification of staphylococcal biofilm maturity

**DOI:** 10.1016/j.bioflm.2025.100283

**Published:** 2025-05-02

**Authors:** S.C.J. van Dun, R. Knol, A.S. Silva-Herdade, A.S. Veiga, M.A.R.B. Castanho, P.H. Nibbering, B.G.C.W. Pijls, A.M. van der Does, J. Dijkstra, M.G.J. de Boer

**Affiliations:** a Leiden University Center for Infectious Diseases (LUCID), Laboratory of Infectious Diseases, Leiden University Medical Center, Leiden, the Netherlands; b Faculdade de Medicina, Universidade de Lisboa, Lisboa, Portugal; c Gulbenkian Institute for Molecular Medicine, Lisboa, Portugal; d Human Health Vision, Zwolle, the Netherlands; e Department of Orthopaedics, Leiden University Medical Center, Leiden, the Netherlands; f PulmoScience Lab, Department of Pulmonology, Leiden University Medical Center, Leiden, the Netherlands; g Division of Image Processing, Department of Radiology, Leiden University Medical Center, Leiden, the Netherlands

**Keywords:** Biofilm, Classification, Atomic force microscopy, *Staphylococcus aureus*, Machine learning algorithm

## Abstract

An increasing incidence of device-related, biofilm-associated infections has been observed in clinical practice worldwide. *In vitro* biofilm models are essential to study these burdensome infections and to design and test potential new treatment approaches. However, there is considerable variation in *in vitro* biofilm models, and a generally accepted systematic description of biofilm maturity – apart from incubation time – is lacking.

Therefore, we proposed a scheme comprised of 6 different classes based on common topographic characteristics, *i.e.*, the substrate, bacterial cells and extracellular matrix, identified by atomic force microscopy (AFM), to describe biofilm maturity independent of incubation time. Evaluation of a test set of staphylococcal biofilm images by a group of independent researchers showed that human observers were capable of classifying images with a mean accuracy of 0.77 ± 0.18. However, manual evaluation of AFM biofilm images is time-consuming, and subject to observer bias. To circumvent these disadvantages, a machine learning algorithm was designed and developed to aid in classification of biofilm images.

The designed algorithm was capable of identifying pre-set characteristics of biofilms and able to discriminate between the six different classes in the proposed framework. Compared to the established ground truth, the mean accuracy of the developed algorithm amounted to 0.66 ± 0.06 with comparable recall, and off-by-one accuracy of 0.91 ± 0.05. This algorithm, which classifies AFM images of biofilms, has been made available as an open access desktop tool.

## Highlights

• Staphylococcal biofilm classification possible based on specific characteristics.
• Classification scheme applicable for (in)experienced human researchers.
• Deep learning algorithm developed for automated biofilm classification.
• The algorithm classifies biofilm with similar accuracy to human researchers.
• Open access tool designed from algorithm available for research.

## 1 Introduction

Biofilms are multicellular bacterial communities in a self-produced extracellular matrix, typically found in almost every part of nature. Bacterial biofilms are an important contributor to global health problems due to their resistance to antibiotics, to the host's immune system, and to other environmental stressors [1]. The National Institute of Health (NIH) states that biofilm formation is associated with 65 % of all microbial diseases and with 80 % of chronic infections [2]. To improve treatment of these infections, there is a need for reliable, well-characterized *in vitro* biofilm-associated infection models. At present, there is a considerable variation between *in vitro* biofilm models in technical set-up, biological input and read-outs. Besides the obvious differences in bacterial strains, culture media and substrates, one of the main variables is the incubation time. Often, biofilm maturity is directly related to incubation time. Together, these variations result in studies reporting on early, immature biofilms, formed within 4 h after start of culture up to 24h, or on late, mature biofilms at 24 h up to 7 days [[3], [4], [5]]. Atomic force microscopy (AFM) imaging showed substantial differences in complexity and maturity, *i.e.,* bacterial cell density, extracellular matrix and visible substrate of staphylococcal biofilms [[6], [7], [8], [9], [10], [11]]. This indicates that defining the maturity of biofilms based on incubation time may not always result in a consistent model. A uniform, unbiased classification system based on biofilm characteristics may provide a more accurate approach to define biofilm maturity. In recent years, machine learning algorithms have demonstrated excellent results in image analysis [[12], [13], [14], [15], [16]]. Since manual evaluation and classification of AFM images is laborious and subjective, a machine learning algorithm could help overcome these disadvantages. We hypothesized that such an algorithm could aid in the detection and classification of biofilms, by taking defined characteristics, e.g., substrate, bacterial cells and presence of extracellular matrix into consideration. This approach was tested for scanning electron microscopy images, where self-supervised deep learning methods were used to detect bacterial cells and byproducts [17]. Software tools such as BiofilmQ [18], BioFilmAnalyzer [19], and COMSTAT [20], have been developed to aid in the mere description of biofilm characteristics and measurements of geometric properties. These methods have shown promise for classifying characteristics within biofilms. However, there is a lack of methods that provide classification of biofilm maturity as a whole, based on beforementioned characteristics. In the current study, this characteristic-based approach of biofilm classification was incorporated. AFM analysis provided highly detailed images of biofilms including these characteristics, which were needed for classification of biofilm maturity. Applying machine learning to these images could provide an unbiased analysis tool for biofilm maturity. Therefore, the aim of this study was to propose a framework that classifies staphylococcal biofilms based on typical biofilm characteristics identified on AFM images, and ultimately design and train a machine learning algorithm to automate this classification scheme.

## 2 Materials and methods

**Fig. 1:**
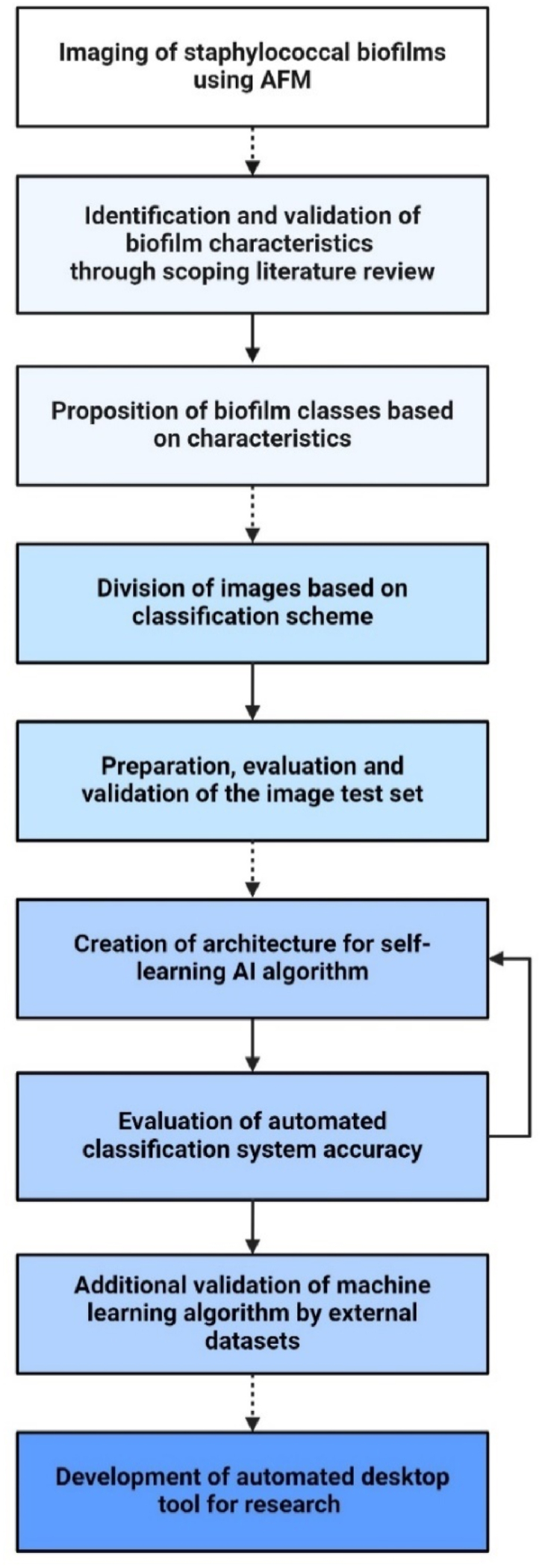
Flowchart of biofilm image classification; from initial biofilm imaging with atomic force microscopy to the development and training of an automated classification system using a machine learning algorithm.

Prior to the development of a machine learning algorithm, consecutive steps were undertaken to establish the anticipated biofilm classification system (Fig. 1). In this section we describe these steps and the development of the corresponding machine learning algorithm.

### 2.1 Biofilm imaging using atomic force microscopy

#### 2.1.1 In vitro implant-associated biofilm model

Two titanium alloys, i.e., medical grade 5 titanium-7 % aluminium-6 % niobium (TAN; ISO 5832/11) discs (diameter 5 mm, height 1.5 mm) and medical grade 5 titanium-6 % aluminium-4 % vanadium (TAV; ISO 5832/3, Braun) discs (diameter 4 mm, height 1.5 mm), were used. All discs were prepared to specifically fit into 96-well plates. Preparation of bacterial suspensions of *S. aureus* LUH14616 and corresponding 24-h (early) and 7-day (late) biofilm culture was based on a validated *in vitro* biofilm model, previously used in different studies [6,21]. Prior to imaging, staphylococcal biofilms were fixed with 0.1 % (v/v; in MilliQ) glutaraldehyde for 4 h at room temperature and samples were left to dry overnight after removing the fixative. After fixation and drying, discs with biofilms were stored at 4 °C prior to imaging with AFM.

#### 2.1.2 Imaging by atomic force microscopy

AFM images of biofilms on implant material discs were acquired using a JPK NanoWizard IV (Berlin, Germany), with an integrated up-right microscope (JPK “TopViewOptics” with Navitar long distance zoom lens) (Fig. S1). Measurements were performed in ambient conditions and in intermittent contact mode, or AC mode, using uncoated silicon ACL cantilevers from AppNano (Mountain View, CA, USA) with typical resonance frequencies of 160–225 kHz, a spring constant of 36–90 N/m and average nominal tip radius of 6 nm. Scan speeds ranged between 0.2 and 0.4 Hz. Scans of 5 μm × 5 μm were taken to obtain detailed images of implant material and biofilm surfaces. For this, 1 individual *S. aureus* cell has a size 1.0 ± 0.5 μm. The captured images were processed using JPKSPM Data Processing software version 6.1.191 (JPK BioAFM, Bruker Nano GmbH, Berlin, Germany).

### 2.2 Class definition and image annotation

Manual screening of AFM images led to the identification of three commonly present characteristics; 1) visible implant material used as a substrate for biofilm formation, 2) bacterial cell coverage, and 3) presence of extracellular matrix (ECM). Initially, to determine what fraction of an image had one or multiple characteristics, a grid of 10 × 10 same-size squares was used to divide each image in 100 individual fractions. Images were then scored by checking each square for the individual characteristics and calculating a percentage for each characteristic. This allowed for separation of images based on percentages. Based on the presence or absence of these characteristics in corresponding percentages, six biofilm classes (0–5) were defined (Table 1).

**Table 1:** Definition of biofilm classes based on presence of defined characteristics. Percentages indicate the ratio of squares in a ten-by-ten framework that have a specific characteristic. N.I.: not identifiable.

Biofilm class	Characteristics		
	Implant material	Bacterial cells	Extracellular matrix
0	100 %	0 %	0 %
1	50–100 %	0–50 %	0 %
2	0–50 %	50–100 %	0 %
3	0 %	50–100 %	0–50 %
4	0 %	0–50 %	50–100 %
5	0 %	N.I.	100 %

**Fig. 2:**
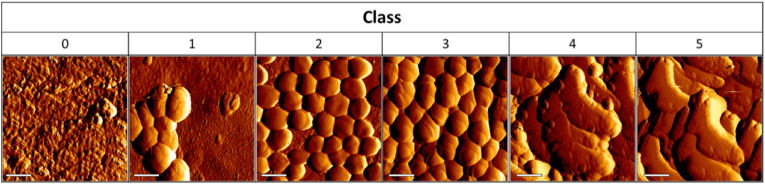
Exemplary AFM images (5 μm × 5 μm) of the various classes of biofilm maturity, identified based on distinct characteristics. Scale bars indicate 1 μm. 1 individual *S. aureus* cell has a size 1.0 ± 0.5 μm.

Class 0 images only consist of the implant material substrate without visible bacterial cells or ECM. The different classes show increased coverage of the substrate in the lower classes and further structural changes through presence and coverage bacteria by extracellular matrix in the higher classes, as indicated by the percentual differences between classes in Table 1 and the corresponding images in Fig. 2. This corresponds to the model of biofilm formation where cell clustering and microcolony formation are common in early stages, and with ECM build-up in later stages. AFM images that were initially taken as part of a previous study [6] were revisited and classified based on Table 1. Examples of each class can be observed in Fig. 2. The annotated images were used as the ground truth for the algorithm to learn and train its parameters. The ground truth can be defined as the reality one wants to recreate with a supervised machine learning algorithm.

### 2.3 Ground truth and inter-observer variability assessment

A small test set of 15 images was taken apart from the annotated images, in which each class was represented at least twice. This test set was then evaluated by seven independent researchers, without prior knowledge or experience with AFM biofilm images. This was done to assess whether the expert-defined ground truth could be interpreted as such by unbiased individuals, and to assess potential inter-observer variability. Observers received a short explanation on how to approach the classification (Supplementary material B). The evaluation was performed in the same manner as described in section 2.2, with the 10 × 10 grid scoring, checking each square for the individual characteristics and calculating a percentage for each characteristic.

### 2.4 Machine learning algorithm design

#### 2.4.1 Dataset preparation and augmentation

From previous AFM biofilm images [6], a dataset was created consisting of 138 unique biofilm images. The distribution of annotated images over the six classes of the classification scheme was uneven. Table 2 shows the number of images for each class. The original AFM images have a resolution of 512 × 512 pixels. To account for the uneven distribution over the various classes, a weighting scheme was used to account for updates to the weights within the network. In this way, the overall accuracy was impacted less per image for classes with more images. For each class, five images were used for the test set and all remaining images were included in the training set. Different data augmentation techniques were applied to enlarge the training set, commonly used for this kind of machine learning models [22]: image rotation, splitting and recombination. Augmented images were generated by applying rotations in 90-degree steps or increasing/decreasing the brightness of each image in small steps. Furthermore, “artificial” images were created by splitting up and recombining original images. For each class, all images were split into four equal segments. Four random sections were concatenated to form a new image. A ratio of four artificial images to each original was used, with the aforementioned augmentations being applied to these images as well. Through these combined approaches, 2700 images were created for training. The rotational augmentations were also applied to the test data. This augmentation is equivalent to the physical rotation of the biofilm sample before measuring it with AFM and visualizing it. Therefore, it provided additional data that was valid for testing purposes, yielding a test set of 120 images, distributed evenly over the classes. It is important to note that even though data augmentation can provide a positive effect on the quality of a trained model, augmented versions of the original images do not fully reflect the true diversity of images that could be obtained from new biofilm samples. The size of the original dataset still imposes fundamental limits to the capabilities of the model.

**Table 2:** The number of original and augmented images for each class and the number of images in the test and training splits of the dataset.

Class	Test		Training		
	Original	Augmented	Original	Artificial	Augmented
0	5	20	12	60	300
1	5	20	8	40	200
2	5	20	14	70	350
3	5	20	15	75	375
4	5	20	39	195	975
5	5	20	20	100	500

**Total**	30	120	108	540	2700

#### 2.4.2 Architecture of deep learning artificial intelligence algorithm

Since a small original dataset was used, a transfer learning approach was chosen [23]. Initially, different architectures were considered: ResNet [24], EfficientNet [25], VGG [26], and Xception [27]. Based on preliminary experiments, the Xception architecture was chosen, which consists of 36 depth-wise separable convolutional layers suitable for feature extraction from image data. This architecture was pre-trained on the ImageNet dataset [28]. ImageNet contains millions of images of natural subjects, such as animals and vehicles. While the images in the study domain are distinct from the ImageNet dataset, the knowledge embedded in the pre-trained weights still provided a large increase in performance compared to a model trained from scratch on only the data of this study. The Xception feature extractor was extended with a fully connected layer of 64 neurons, followed by a dropout layer with a dropout strength of 0.25. This layer was then fed into the final classification layer. After preliminary testing, the biofilm analysis was set up as classification rather than (ordinal) regression. Hence, the final layer had six neurons, one for each class. The input images were resized to 128 × 128 pixels, as it increased the model's predictive quality. The model was trained for 20 epochs with a learning rate of 5e-5 and cross-entropy loss function. An epoch is defined as the number of passages the training dataset makes through an algorithm. The learning rate determines how much the neural network weights change within the context of optimization while minimizing the loss function.

#### 2.4.3 Evaluation of the automated classification system

The model was evaluated on three different metrics: accuracy, F1 score, and off-by-one accuracy **(**Eqs. S1-S5**)**. The former two are well-established metrics for classification problems [29], with F1 score serving as a metric for the precision and recall of the algorithm. The off-by-one accuracy was defined as the proportion of predictions that was at most one step removed from the ground truth. This metric was included as most of the model's errors were observed to be off-by-one. Moreover, given that this task can, in practice, be understood as (ordinal) regression, this additional metric was added to emphasize that the model can still make informative predictions, even when they are not fully precise. Additionally, simplified versions of the classification scheme, where classes are merged together, were assessed. This resulted in a fundamentally simpler classification problem. The merging of classes was based on the assumption that these versions of the scheme would provide the most information. Table 3 lists the variant of the classification scheme, noting which classes were merged and the resulting amount of classes. The models that were trained to learn these variants had their final layers modified accordingly. The test sets for the simplified versions were balanced according to the merged classes, meaning that they still had 5 original images per class. Since they have fewer classes in total, a larger fraction of the data was used for training.

**Table 3:** The four different versions of the classification scheme. We note which classes have been merged and the total number of classes this resulted in.

Name	Merged Classes	Total Number of Classes
Standard	–	6
Compressed Start	1–2	5
Compressed End	4–5	5
Compressed Both	1-2; 4-5	4

To evaluate the performance of the algorithm over time, additional measures were taken into account. First, the cross-entropy loss was evaluated to assess potential overfitting of the data. This metric can be defined as the difference between the discovered probability distribution of a classification model and the predicted values of this model. Second, a class confusion matrix was created for the algorithm. This summarizes the class predictions made by a model (columns) against the actual ground truth values (rows) as a normalized fraction, rounded up to a value of 1. In addition to the mean, the minimum and maximum fraction in each cell were included, to indicate the variance between models. Cells along the diagonal indicate correct predictions. The further away from the diagonal, the larger the discrepancy from the ground truth.

#### 2.4.4 Validation of the algorithm using other datasets

To validate the accuracy of the algorithm on external data, we collaborated with the Miguel Castanho Lab, Gulbenkian Institute for Molecular Medicine, in Lisbon. This group used the same protocol for biofilm fixation and AFM imaging as described in this study, with the same *S. aureus* strain LUH14616. Additionally, the Miguel Castanho Lab used the *S. aureus* ATCC 6538 strain for biofilm imaging. The former images were used for validation of the experimental procedure. The latter images were included into the algorithm to assess whether our classification was suitable for multiple *S. aureus* strains. In addition, since *S. aureus* is not the only causative microorganism for biofilm-associated PJIs, biofilms of the coagulase-negative *Staphylococcus epidermidis* LUH15408 strain were imaged with AFM for additional validation of the algorithm.

#### 2.4.5 Test-retest reliability

To gain a more nuanced understanding of the performance of the algorithm, images from the dataset were revisited and discrepancies from the ground truth were assessed. The off-by-one discrepancies were taken from the dataset and evaluated on over- or underestimation of classes. In addition, images that were classified with discrepancies were taken from the dataset and reevaluated on origin and quality.

## 3 Results

### 3.1 Ground truth and inter-observer variability assessment

The expert-defined ground truth was assessed by seven inexperienced, independent observers, before the annotated images were used as input for the algorithm. From their classification of 15 annotated images, a mean accuracy of 0.77 ± 0.18 was calculated. Individually assigned images per researcher are shown in Table S1. Assessment of each individual classification indicated that most discrepancies from the ground truth were close to the actual class. Therefore, another metric was considered where discrepancies of 1 class higher or lower (off-by-one) than the ground truth were acceptable. This assessment resulted in an off-by-one accuracy of 0.90 ± 0.15. Together, these metrics indicated considerable inter-observer variability.

### 3.2 Algorithm accuracy performance

Once the model was developed, its accuracy needed to be tested. For this, four methods were compared with results reported as the mean ± standard deviation over 50 independent runs (Table 4 and Fig. S2). For each of these runs, the training and test sets were resampled randomly. Based on the standard version of the classification scheme, the algorithm reached an accuracy of 0.66 ± 0.06 while the simplified versions scored better, up to 0.83 ± 0.06.

**Table 4:** The performance of different versions of the model on the test set, expressed in terms of accuracy, off-by-one accuracy, and F1 score. Reported values are mean accuracy and standard deviations over 50 independent runs.

Method	Accuracy	Off-by-one Accuracy	F1
Standard	0.66 ± 0.06	0.92 ± 0.04	0.65 ± 0.06
Compressed Start	0.74 ± 0.07	0.93 ± 0.04	0.74 ± 0.07
Compressed End	0.74 ± 0.06	0.92 ± 0.05	0.73 ± 0.06
Compressed Both	0.83 ± 0.06	0.91 ± 0.05	0.83 ± 0.07

F1 scores were very similar to the accuracy scores, indicating no significant imbalance between precision and recall. For each version of the model, the off-by-one accuracy was ≥0.91 ± 0.05, indicating that most discrepancies were small, even with the standard amount of classes.

As the different methods of the model showed differences in the evaluated metrics (Table 4), these variations were also applied to the results of external researchers described in 2.2.4. This resulted in differences in the accuracy, with standard deviations remaining large. Comparing these results to the accuracy of the algorithm, similar values were observed in the classification by the independent researchers. However, the standard deviations indicate that the performance of the algorithm was more consistent than the independent human researchers (Table 5).

**Table 5:** The performance of independent researchers (N = 7) divided into the different methods of the classification system, expressed in terms of accuracy and off-by-one accuracy compared to the ground truth. Reported values are mean accuracy and off-by-one accuracy with standard deviation from a test set of 15 images.

Method	Accuracy	Off-by-one Accuracy
Standard	0.77 ± 0.18	0.90 ± 0.15
Compressed Start	0.74 ± 0.18	0.88 ± 0.16
Compressed End	0.78 ± 0.19	0.90 ± 0.16
Compressed Both	0.74 ± 0.20	0.87 ± 0.17

As an additional measure, the cross-entropy loss was computed on the training and test set after each epoch for the standard (6-class) classification scheme (Fig. S3A). The results are averaged across the 50 independent runs, and the margin indicates the full spread of the data. Based on the full distribution, it became apparent that in some cases, the test loss stabilizes, meaning there is no overfitting. Within the span of 20 epochs, overfitting did not cause a collapse in accuracy (Fig. S3B), which showed a stable convergence.

### 3.3 Class confusion assessment

**Fig. 3:**
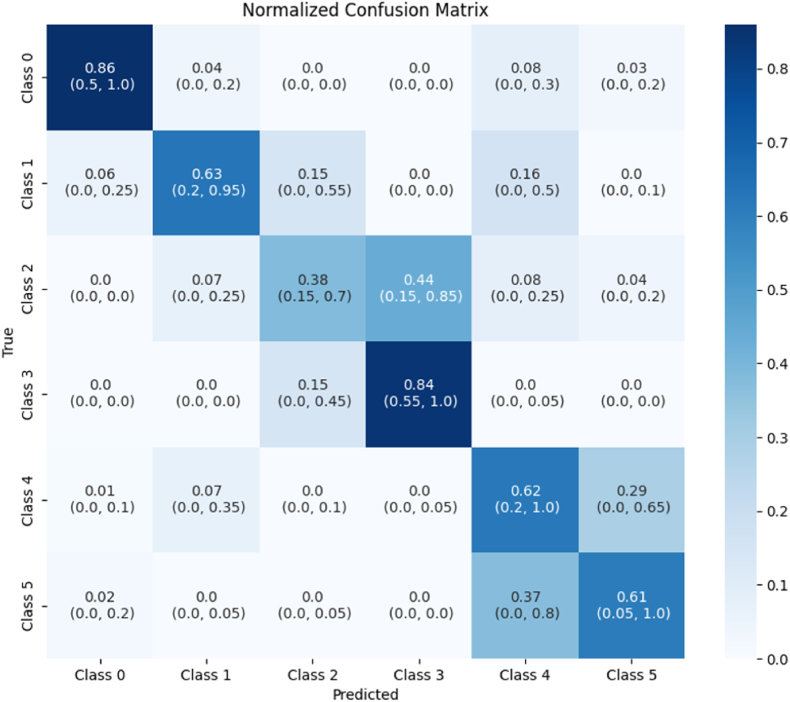
A confusion matrix of the predictions of 50 independent runs, normalized by row. It shows the fraction of images of each true label that was predicted as belonging to a certain class. Below the average, the maximum and minimum of all runs are shown. The cells along the diagonal are the correct predictions.

To gain insight into the erroneous classifications by this algorithm, results of misclassified images were assessed over 50 independent runs. From this, a class confusion matrix was created (Fig. 3). Most predictions were on, or next to, the diagonal. These predictions are therefore considered correct or off-by-one. Notably, most discrepancies were observed in the top right of the confusion matrix, where instances of class 0 and class 1 were mistakenly predicted to belong to class 4.

### 3.4 Validation of the algorithm

#### 3.4.1 External laboratory assessment

The generalization of the algorithm was assessed using data obtained by the Miguel Castanho Lab at the Gulbenkian Institute for Molecular Medicine in Lisbon, Portugal. This group used the same protocol for biofilm fixation and AFM imaging described in this study, albeit with a different biofilm culture protocol [10]**.** AFM images from *S. aureus* strain LUH14616 were used for validation of the classification model (Table S2). The standard model inference output resulted in an accuracy of 0.56 ± 0.04, with the off-by-one accuracy showing a high value of 0.92 ± 0.03.

#### 3.4.2 Algorithm performance on alternative strains and species

Additionally, images of *S. aureus* ATCC 6538 biofilms were presented to the model to assess the suitability of our classification algorithm for other *S. aureus* strains [10] (Table S2). Running the standard model resulted in an accuracy of 0.47 ± 0.06, with off-by-one accuracy being 0.97 ± 0.02.

**Fig. 4:**
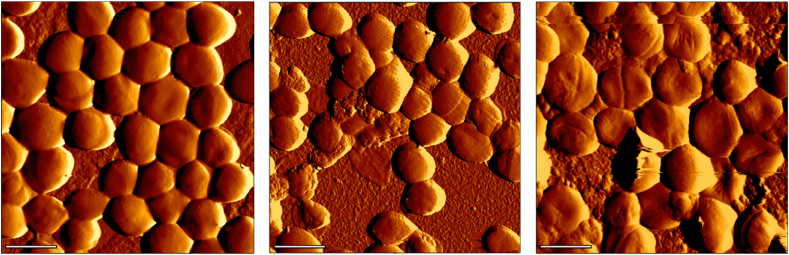
Exemplary AFM images (5 μm × 5 μm) of the *S. aureus* LUH14616 (left), *S. aureus* ATCC 6538 (middle) and S. epidermidis LUH15408 (right). Images have been identified as class 2 based on the described scheme. Scale bars indicate 1 μm. 1 individual *S. aureus* cell has a size of 1.0 ± 0.5 μm.

Since *S. aureus* is not the only causative microorganism for biofilm-associated PJIs, the coagulase-negative *Staphylococcus epidermidis* LUH15408 biofilms were imaged with AFM to be included as an additional validation of the algorithm. Unfortunately, the current model had difficulties with the assessment of the biofilm formed by this other species, as standard and off-by-one accuracy did not exceed 0.26 ± 0.03 and 0.59 ± 0.03, respectively (Table S2). The tested strains had easily identifiable biofilm characteristics and could be manually classified based on the scheme, despite the low accuracy in the algorithm. Examples of biofilms from the different strains within the same class are shown in Fig. 4.

#### 3.4.3 Test-retest reliability

To gain a nuanced understanding of the performance of the model, the percentage of discrepancies where the predicted class was higher or lower than the true value was assessed. This showed that the small off-by-one discrepancies are equally over- and underestimated, *i.e.* 0.51 and 0.49, respectively, indicating limited bias for the model on this aspect. The larger discrepancies, however, were more likely to overestimate the class, with a ratio of 0.77. This corresponded with the higher number of images in the higher classes. Of note, deeper assessment of the classification discrepancies showed that only a relatively small sample of 22 *S. aureus* biofilm images exceeded the off-by-one margin within 50 runs. From these 22 images, 5 were from the external lab validation set, which had lower accuracy overall. Additionally, six images showed artefacts that may have made correct classification more difficult.

### 3.5 Setup of research tool based on classification algorithm

From the developed algorithm, an open access tool was created [https://github.com/Ram9bo/image-classification.git] that allows for public use of the classification system. This tool was successfully tested on laptop and desktop computers with varying processing speeds to assess its useability and accessibility in different systems. It allowed for input of images and even folders of images, from which the tool generates an Excel spreadsheet data file with the image name and corresponding classification.

## 4 Discussion

Differences in biofilm research methodology have led to considerable variation in the definition of biofilm maturity, thus increasing complexity in the interpretation, extrapolation and comparison of results. In this study, we defined a classification system based on common biofilm characteristics rather than on incubation time, that allowed for assignment of staphylococcal biofilms to different phases of maturity. In addition, the characteristics were used as a basis for a machine learning algorithm for automated classification, for which this study provides a proof-of-concept. This classification system can be used to aid in interpretation of staphylococcal biofilm images by human observers.

Previously, others have applied various forms of artificial intelligence to aid in classification of biofilms, with variable levels of success. For example, Ragi et al. [30], developed a comparable deep learning convolutional network to assess characteristics of biofilms. However, this study made use of *Desulfovibrio alaskensis* G20, a sulfate-reducing bacterial species, in the context of industrial corrosion, which is not clinically relevant. Additionally, their dataset consisted of only 66 scanning electron microscopy (SEM) images and reported accuracies ranging from 60 % to 75 %. Another study by Abeyrathna et al. [17], created self-supervised learning models for classification of SEM biofilm images from the same strain used by Ragi et al. [30]. Abeyrathna et al. applied a similar set of characteristics, e.g.*,* byproduct, cell and surface, reporting accuracies ranging from 62 % up to 96 %. However, this study lacks further classification of biofilms based on the identified characteristics, and also used a clinically irrelevant bacterial species. In addition, their algorithm was built on an even smaller dataset of 7 SEM images. Andrade et al. [31], developed a segmentation approach for biofilms in intra-oral photographs. This study focused on photographs of dental plaque biofilm as a whole, without specific characteristics or bacterial species, in comparison to photographs with a disclosing agent. They build an algorithm based on 480 images, with a frontal, right and left view from 160 patients, with the ImageNet database as a backbone. This study reported an accuracy of 91.8 %, but with an F1 score of 60.6 %. The authors stated that the moderate F1 score and sensitivity were associated with the complexity of labelling dental biofilm on photographs, due to undefined and amorphous borders. Since our study used detailed AFM images, this issue did not occur here. Considering the range of findings in previous studies, our approach can be considered novel, since AFM biofilm images have not yet been subjected to characterization and corresponding classification using artificial intelligence. Furthermore, the accuracy and F1 scores from this proof-of-concept study were comparable to the results of biofilm image classification by human observers.

Unfortunately, the current study suffers from a limited number of images. Generally, large numbers of images are required to train algorithms based on a deep neural network structure. However, as culturing of these biofilms takes hours or days, this limited the volume of the data. Additionally, the imaging with AFM and the corresponding analysis and annotation of images is a time-consuming method. Due to efforts to increase the size of the dataset, using augmentation methods, our study included 2700 images from the original 136 images. It is important to note again that the augmented images do not represent the actual variability between biofilm images. In this study, these augmented images broaden the selection from which the algorithm can learn and use information, without the addition of real-world AFM imaging samples. However,. this number of images proved insufficient to reach an accuracy of more than 70 % in the standard model. Transfer learning models based on other databases helped us prepare a data-performance correlation, showing that comparable models require at least a ten-fold of images to increase the accuracy significantly (data not shown). Another limitation of the current data set could be related to a skewing towards relatively easy or relatively difficult to classify images. Additionally, certain features of biofilm images may be underrepresented or missing entirely. Provided that some of our images were generated in two different labs, and every experiment may show differences in biofilm formation, some of the variance in the true distribution has likely been captured. However, the number of images per class was skewed to the more mature phases of biofilm formation (class 4/5). After reevaluation, the off-by-one accuracy showed no bias in the amount of mistakes between the lower and higher end of the classification. The creation of a much larger dataset is a logical next step to turn the model from a proof-of-concept for classification into a tool with higher accuracy and smaller margin of error. To create a more robust algorithm, more images – mostly for the lower classes – are required to obtain a more normalized distribution of the different classes of biofilm images.

Importantly, the current algorithm already reached an accuracy comparable to a group of independent researchers. Additionally, the algorithm was built based on images of one *Staphylococcus aureus* isolate, *i.e.,* LUH14616. To assess its widespread applicability, more images of other isolates need to be added to the dataset, since variation between isolates could have an effect on the accuracy, as indicated by the results for *S. aureus* ATCC strain 6538 (section 3.4.1, 3.4.2). If this proves effective, the model could be further expanded to other staphylococcal species like the *S. epidermidis* LUH15408 in section 3.4.2, and potentially even adapted to a model for other groups of biofilm-forming microorganisms that follow a similar biofilm maturation pattern. Overall, it is important to note that the inclusion of the additional *S. aureus* and *S. epidermidis* strains was an exploratory aspect of this study, to assess more broad applicability. Despite the limited accuracy, these results were included to gain a more nuanced understanding of the algorithm.

The confusion matrix showed that there were discrepancies between the classes predicted by the algorithm and the ground truth assigned to the images. Most discrepancies were observed in images from class 0. Here the models had some difficulties in classifying the images, with some assigning class 4/5 to these images. These discrepancies were also observed in the human test-set classification. This indicates that these images have some overlap in their characteristics, but also that the model is comparable in its function to manual scoring. The discrepancies can only be reduced with larger datasets and better manual classification.

One of the main advantages of the model is the aspect of time. It can classify up to hundreds of images within minutes, whilst human scoring could take several minutes to classify a single image by 10 × 10 square fragmentation. This way, the algorithm can be opened whilst imaging with AFM and one would be able to classify an image immediately after generating it, to gain insight in the maturity of the cultured biofilm. It also removes the aspect of human variation, since the algorithm can score images based on specifically defined characteristics, without having the possible variations and misclassification when each square is scored manually. Importantly, the algorithm removes bias in classification based on the prior knowledge on culture conditions and incubation time, as the program does not have this information input.

As a future prospect, the current biofilm classification tool could aid in understanding the effects of antimicrobial treatment modalities on bacterial biofilms*.* For example, the decrease in bacterial cells or loss of well-defined characteristics if the extracellular matrix is disrupted, could be assessed, expanding its applicability in research. These antimicrobial-induced changes to biofilms have been studied using AFM before [6,10,32,33]. Additionally, imaging of actual infected patient material such as fixation screws, k-wires and plates could provide an understanding of how biofilms are formed and maintained *in vivo*, and give insight into their maturity state*.* In this way, an association could be made between the biofilm maturity and severity of infection and treatment outcomes, even making the algorithm useful in a clinical setting.

In conclusion, this study describes a staphylococcal biofilm classification method using specific characteristics, *i.e.*, I) visible implant material used as a substrate for biofilm formation, II) bacterial cell coverage, and III) presence of extracellular matrix (ECM), identified by AFM imaging. This classification was validated by external researchers without prior experience with AFM biofilm images, indicating recognizable patterns within the various classes of biofilm images and clear differences between these classes. This scheme and the corresponding biofilm characteristics were used as input to design a deep learning model. This algorithm was developed and trained on our AFM image dataset, showing an accuracy of ∼70 % and an off-by-one accuracy of >90 % with good reproducibility, showing small margins of error. The overall accuracy of the model and the corresponding tool [https://github.com/Ram9bo/image-classification.git] was comparable to manual, human classification, in considerably less processing time. Together, these findings form a proof-of-concept for the classification scheme and the corresponding machine learning algorithm.

## CRediT authorship contribution statement

**S.C.J. van Dun:** Writing – review & editing, Writing – original draft, Visualization, Validation, Resources, Project administration, Methodology, Investigation, Formal analysis, Data curation, Conceptualization. **R. Knol:** Writing – review & editing, Writing – original draft, Validation, Software, Methodology, Formal analysis, Data curation. **A.S. Silva-Herdade:** Writing – review & editing, Validation, Methodology, Investigation, Data curation. **A.S. Veiga:** Writing – review & editing, Validation, Methodology, Data curation. **M.A.R.B. Castanho:** Writing – review & editing, Validation, Methodology, Data curation. **P.H. Nibbering:** Writing – review & editing, Supervision, Project administration, Conceptualization. **B.G.C.W. Pijls:** Writing – review & editing, Supervision, Conceptualization. **A.M. van der Does:** Writing – review & editing, Supervision, Project administration. **J. Dijkstra:** Writing – review & editing, Supervision, Conceptualization. **M.G.J. de Boer:** Writing – review & editing, Supervision, Resources, Project administration, Funding acquisition, Conceptualization.

## Declaration of competing interest

The authors declare that they have no known competing financial interests or personal relationships that could have appeared to influence the work reported in this paper.

## Data Availability

Data will be made available on request.
