# Supplementary material for: Machine learning assisted classification of staphylococcal biofilm maturity
Source: Biofilm. 2025 May 2;9:100283. doi: 10.1016/j.bioflm.2025.100283 (PMC12127609; doi:10.1016/j.bioflm.2025.100283)
Supplement: Multimedia component 1 [file mmc1.pdf]

Appendices

A. Schematic overview of atomic force microscopy imaging

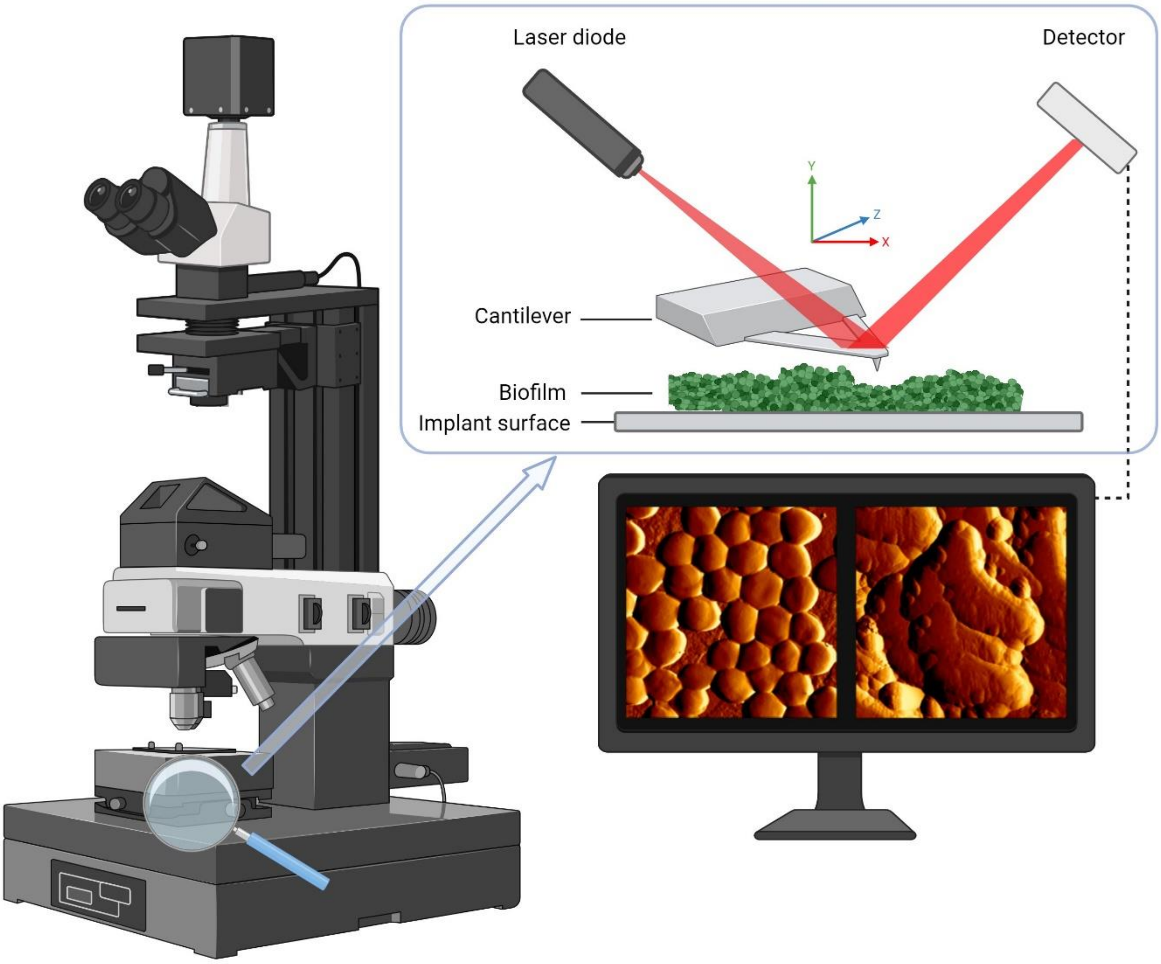

Figure S1: Setup of atomic force microscope (left) and schematic overview of the technique (right) where a diode emits a laser onto a cantilever, moving in three directions over the biofilm surface. The cantilever reflects the laser into a detector, providing frame-by-frame images of the surface. (created with Biorender.com)

B. Introductory text to inform independent observers

In the attachments, there is a test set of 15 AFM biofilm images. To get an understanding of the concept of this classification, there is some important points that need to be noted. Each image is 5 µm x 5 µm in size (1 individual *S. aureus* cell has a size of 1.0 ± 0.5 µm). Each image has a 10x10 raster overlay (100 squares total). Each square can be scored for the following: I) Substrate/implant material on which the biofilm is cultured; II) *Staphylococcus aureus* bacterial cells and III) Extracellular matrix. Each class is represented in this test set and images will be in a random order, to be judged independently. Each square holds 1% of the total image. Of note, some tiles may hold multiple characteristics, i.e., part of a bacterial cell and underlying substrate, the total percentage may exceed 100%.

C. Equations for calculation of algorithm metrics

(S1) $$Accuracy = \frac{TP+TN}{TP+TN+FP+FN}$$

(S2) $$Off-by-one\ Accuracy = \frac{(TP\pm 1)+(TN\pm 1)}{TP+TN+FP+FN}$$

(S3) $$Precision = \frac{TP}{TP+FP}$$

(S4) $$Recall = \frac{TP}{TP+FN}$$

(S5) $$F1-Score = \frac{2\times Precision\times Recall}{Precision+Recall}$$

Definitions:

TP: True positive; the number of correctly classified positive samples

TN: True negative; the number of correctly classified negative samples

FP: False positive; the number of samples incorrectly classified as positive

FN: False negative; the number of samples incorrectly classified as negative

D. Biofilm classification by independent human observers

Table S1: Classification of a biofilm image test set by independent researchers based on previously defined characteristics, with discrepancies between the ground truth and classification per person.

Ground truth	Test person							Result discrepancies		
class	1	2	3	4	5	6	7	-	1	>1
**0**	5	0	0	0	4	4	0	4	0	3
**0**	5	0	1	0	0	5	1	3	2	2
**0**	1	0	0	5	0	5	4	3	1	3
**1**	1	2	1	1	1	1	1	6	1	0
**1**	1	1	1	1	1	1	1	7	0	0
**2**	2	3	2	2	2	2	2	6	1	0
**2**	2	2	2	2	2	2	2	7	0	0
**3**	2	0	3	3	2	2	2	6	0	1
**3**	3	3	3	3	4	3	3	6	1	0
**4**	4	4	4	5	4	5	3	5	2	0
**4**	4	4	4	5	4	4	3	5	2	0
**4**	4	4	4	5	5	4	5	4	3	0
**5**	5	0	5	5	4	5	5	5	1	1
**5**	5	5	5	5	2	5	5	6	0	1
**5**	5	0	4	5	5	5	5	5	1	1

E. Algorithm accuracy performance

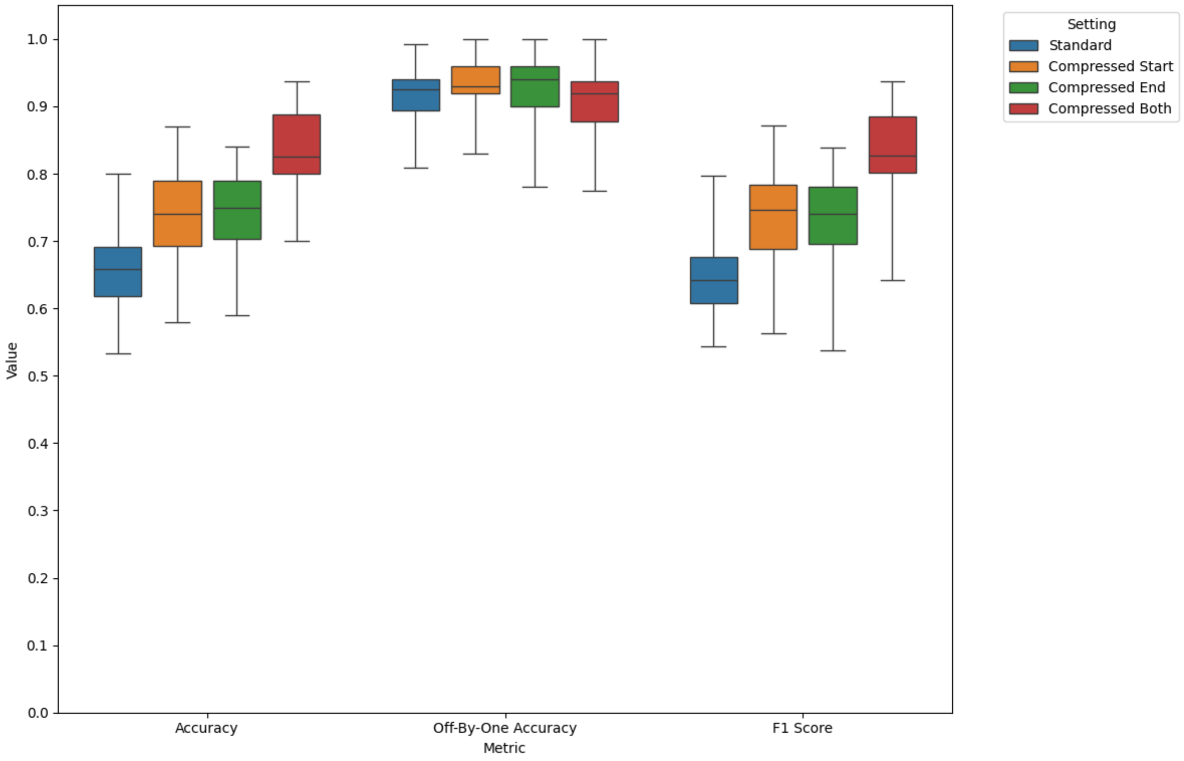

Figure S2: The performance of different versions of the model on the test set, expressed in terms of accuracy, off-by-one accuracy, and F1 score over 50 independent runs. Boxplots show median values with the limits of the 1st and 3rd quartile as the box, with minimum and maximum values indicated by the error bars. Standard model is shown in blue; compressed start in orange; compressed end in green and compressed both in red.

F. Cross-entropy of the algorithm

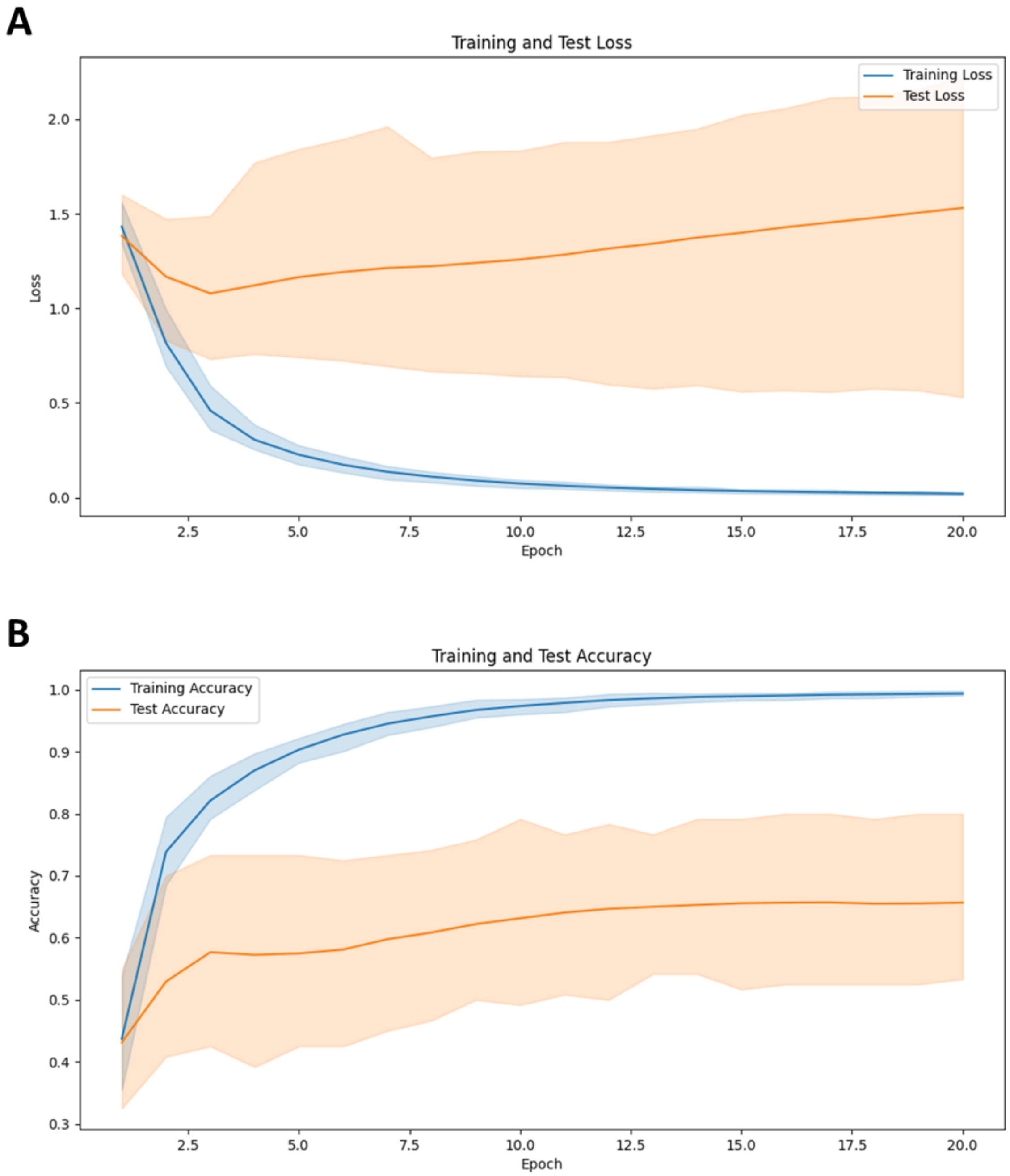

Figure S3: A) line plot of the training (blue) and test (orange) loss per epoch, averaged over 50 independent runs. Margins indicate the full spread of loss across all runs. B) line plot of the training (blue) and test (orange) accuracy per epoch, averaged over 50 independent runs. Margins indicate the full spread of loss across all runs.

G. Algorithm performance on alternative strain datasets

Table S2: The performance of different versions of the model on the additional test sets of alternative strains, expressed in terms of accuracy, off-by-one accuracy, and F1 score. Reported values are mean accuracy and standard deviations over 50 independent runs.

Test set	Method	Accuracy	Off-by-one accuracy	F1
S. aureus LUH14616 (external)	Standard	0.56 ± 0.04	0.92 ± 0.03	0.57 ± 0.03
	Compressed Start	0.58 ± 0.05	0.89 ± 0.03	0.60 ± 0.05
	Compressed End	0.58 ± 0.04	0.92 ± 0.03	0.59 ± 0.03
	Compressed Both	0.62 ± 0.03	0.89 ± 0.03	0.63 ± 0.04
S. aureus ATCC6538	Standard	0.47 ± 0.06	0.97 ± 0.02	0.45 ± 0.06
	Compressed Start	0.55 ± 0.05	0.98 ± 0.02	0.52 ± 0.06
	Compressed End	0.45 ± 0.05	0.97 ± 0.02	0.43 ± 0.05
	Compressed Both	0.53 ± 0.04	0.98 ± 0.02	0.50 ± 0.04
S. epidermis LUH15408	Standard	0.26 ± 0.03	0.59 ± 0.03	0.33 ± 0.03
	Compressed Start	0.25 ± 0.03	0.59 ± 0.02	0.32 ± 0.04
	Compressed End	0.36 ± 0.03	0.63 ± 0.02	0.42 ± 0.03
	Compressed Both	0.34 ± 0.02	0.62 ± 0.02	0.40 ± 0.03
